# Proceedings of the 7th Series of Seminars on Advances in Apomixis Research

**DOI:** 10.3390/plants10030565

**Published:** 2021-03-17

**Authors:** Viviana Echenique, Daphné Autran, Olivier Leblanc

**Affiliations:** 1 Centro de Recursos Naturales Renovables de la Zona Semiárida (CERZOS-CCT Bahía Blanca—CONICET), Bahía Blanca 8000, Argentina; 2 Departamento de Agronomía, Universidad Nacional del Sur (UNS), Bahía Blanca 8000, Argentina; 3 DIADE, Université de Montpellier, CIRAD, IRD, Montpellier, France; daphne.autran@ird.fr

**Keywords:** apomixis, seed development, flowering plants, regulation of gene expression, H2020-MSCA-RISE-2020

## Abstract

These proceedings contain the abstracts for the presentations given at the 7th biennial Seminars on Advances in Apomixis Research, held virtually on 2–3 and 9 December 2020. The first day hosted the kick-off meeting of the EU-funded Mechanisms of Apomictic Development (MAD) project, while the remaining days were dedicated to oral presentations and in-depth exchanges on the latest progress in the field of apomixis and plant reproductive biology research.

## 1. Introduction

**Figure 1:**
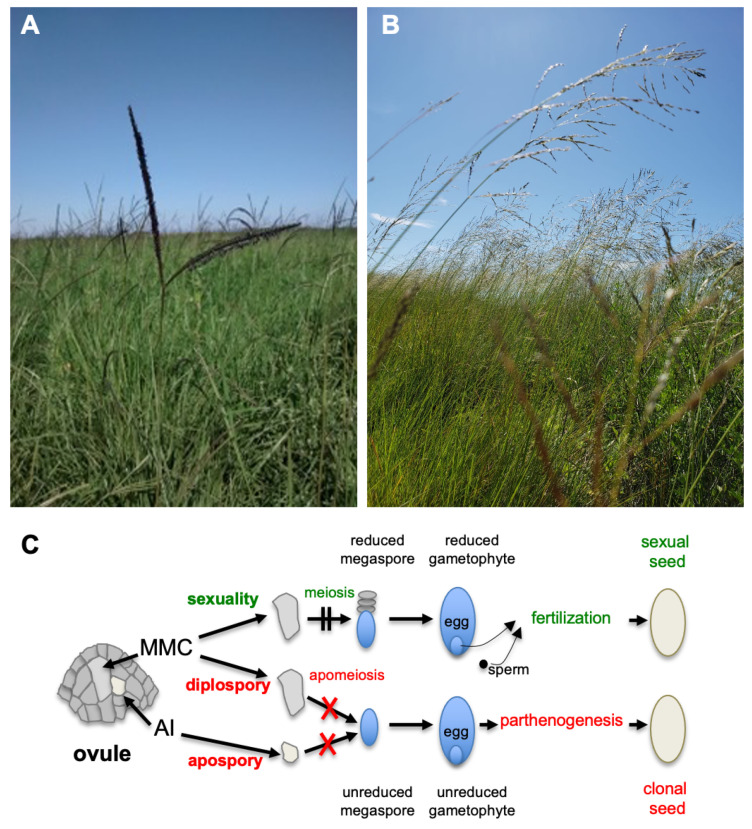
Argentinean apomictic grasses and apomixis reproductive pathways. (**A**) Pastures of *Paspalum notatum*, an example of aposporous plant. (**B**) *Eragrostis curvula*, an example of diplosporous plant. (**C**) Schematic representations of sexual and apomictic reproductive pathways. In sexuality (top), a single cell of the ovule enters the germline differentiation program, the so-called megaspore mother cell (MMC). The MMC undergoes meiosis, hence forming a reduced (haploid) functional spore (megaspore), that differentiates into a reduced gametophyte harboring the female gamete (egg). After fertilization of the egg by a sperm cell, a sexual seed is formed. In apomictic pathways, functional unreduced female gametophytes are formed (apomeiosis), and unreduced eggs develop into maternal embryos in the absence of fertilization (parthenogenesis), resulting in seeds containing clones of the mother plant. In diplospory (middle), meiosis in the MMC fails. The unreduced female gametophyte then arises from an unreduced megaspore. In apospory (bottom), one or more somatic ovule cells differentiate into aposporous initials (AIs), the precursor cells of unreduced female gametophytes.

A staggering increase in food production will be needed to feed a population of nearly 10 billion people in the near future. The growing consensus is that global food demand will double during the next 50 years, representing twice as much as the total amount produced since the beginning of agriculture 10,000 years ago [1]. The need for robust, high-yielding crops adapted to diverse and changing environments is a cornerstone of sustainable development that strongly rely on new agriculture practices and molecular technologies [2,3]. To achieve this, plant reproductive biology represents a challenging research area. Understanding the intricate networks controlling germline development, double fertilization, and seed formation is crucial for delivering innovative approaches to breeders and farmers [4,5]. In this context, the harnessing of apomixis, a natural phenomenon allowing the formation of maternal embryos within seeds [6,7], is among the most promising approaches [8]. Breeding processes typically consist of large-scale designs aimed at exploring the phenotypic value of thousands of genetically fixed (i.e., reproducible) allelic assortments. However, the biology of crop species, including sexual reproduction, renders the implementation of these operations complex, time-consuming, and usually expensive (e.g., [9]). In contrast, any apomictic genotype breeds true, including that of F1 hybrids perpetuating hybrid vigor (or heterosis) over generations [10]. The fixation of heterosis through apomixis would greatly facilitate the deployment of hybrid seed technologies in autogamous crops such as wheat or rice and reduce costs of hybrid seed production in allogamous crops such as maize [11,12,13]. In addition, installing apomixis in major crops could also accelerate breeding cycles and increase variety turn-over, expand genetic diversity by overcoming meiotic sterility resulting from wide crosses and inter- or high ploidy crosses. Apomixis encompasses numerous developmental paths that fall into adventitious embryony where sexually derived seeds harbor a typical sexual embryo together with maternal embryos directly formed from maternal tissues and gametophytic apomixis, where seeds derive from non-reduced female gametophytes carrying parthenogenetic embryos (Figure 1) [6,14]. Apomixis is usually inherited in a dominant manner through a limited number of loci, but the suppression of recombination in the region bearing these loci suggests a more complex genetic control [6,7,14,15,16]. Nevertheless, numerous works indicate the intimacy of these pathways with the factors controlling female germline specification and development, and embryo formation within ovules of sexual plants [17,18]. Unfortunately, despite decades of research, neither transfer attempts from wild apomictic relatives into maize [19] or pearl millet [20] nor the engineering of apomictic reproduction in a sexual crop [13] have yielded the expected results and the establishment of functional apomixis in major crops remains eagerly awaited.

To gain a deeper understanding of the genetic and molecular bases of gametophytic apomixis from natural systems, an international research network was initiated by Argentinian scientists in 2008 at the Centro de Recursos Naturales Renovables de la Zona Semiárida (CERZOS CONICET—Universidad Nacional del Sur, Bahia Blanca, Argentina). Since then, a series of biennial Seminars on Advances in Apomixis Research, has been held in Argentina, under rotating chairing by scientists from CERZOS, the Instituto de Investigaciones en Ciencias Agrarias de Rosario (IICAR CONICET—Universidad de Nacional de Rosario, Santa Fe, Argentina) and the Instituto de Botánica del Nordeste (IBONE CONICET—Universidad Nacional del Nordeste, Corrientes, Corrientes, Argentina). This network has gathered momentum, size, and strength owing to support from the European Union’s Horizon H2020 research and innovation programme through two Marie Skłodowska-Curie grant agreements: Harnessing Plant Reproduction for Crop Improvement (PROCROP, 2014–2018, n° 645674) and Mechanisms of Apomictic Development (MAD, 2020–2024, n° 872417) which kick-off meeting was organized as a one-day session of the 7th Series of Seminars on Advances in Apomixis Research. These proceedings contain a brief report of the MAD kick-off meeting and the abstracts of the presentations given at the 7th Seminars on Advances in Apomixis Research, chaired by Prof. Viviana Echenique from CERZOS-CCT.

## 2. Launch of the Mechanisms of Apomictic Development (MAD) Project

Leblanc O

DIADE, Univ Montpellier, CIRAD, IRD, Montpellier France

The Mechanisms of Apomictic Development (MAD) project that brings together young and experienced scientists from 12 laboratories from Europe, the Americas, and Australia (Table S1), was launched 1 December 2020. This 48-month project consists of a Marie Skłodowska-Curie Research and Innovation Staff Exchange (MSCA RISE) Action funded by the European Union’s H2020 Programme and aimed at supporting international and intersectoral collaboration through the exchange of research and innovation staff. The kick-off meeting was held on 2 December 2020, in conjunction with the 7th Seminars on Advances in Apomixis Research, with a two-session agenda dedicated to partners/work packages presentations and to management issues. Here, a report for the first session is provided, including the summary of the project, an overview of the proposed work packages, and the list of participating institutions and contacts. More information is available from the EU CORDIS portal (https://cordis.europa.eu/project/id/872417 (accessed on 3 February 2021)), and a dedicated website will be set up online in the forthcoming weeks.

### 2.1. Project Summary

Apomixis in plants allows the formation of seeds carrying maternal embryos. While absent in major food crops, it occurs in many plants, including wild relatives of cereals and species of economic interest such as forage grasses and fruit trees. This unique reproductive mode can be achieved through many paths, all involving alterations in the orchestration of the developmental programs underlying sexual reproduction (Figure 1). Since it allows the use of a natural carrier, the seed, for propagating the best genotypes regardless of their constitution, apomixis represents a revolutionary tool for plant breeding programs and for reducing the costs of improved variety seeds. Despite wide-cross breeding programs to introduce the trait in cereals and decades of research using both sexual plant models and apomictic species, apomixis remains an enigma for plant biologists and a long-awaited tool by breeders and farmers. Functional analyses in Arabidopsis and maize have provided valuable molecular information to understand sexual reproduction and, occasionally, to explore alterations yielding phenotypes reminiscent of apomixis. On the other hand, the recent advances in « omics » tools and biotechnologies have opened the route for investigating apomictic species at unprecedented, cellular, and molecular levels. The MAD (Mechanisms of Apomictic Development) project will establish an international, research and training network aiming at contributing significantly to our understanding of key mechanisms involved in redirecting sexuality in plants towards apomixis. It bridges critical knowledge and biological resources recently generated by collaborative efforts in the field of apomixis biology, and novel expertise in plant reproductive biology, biotechnology, and breeding by aggregating new partners. Synergies are expected from three types of complementarities, including the following: biological systems and resources prone to fuel comparative and translational research (Table 1); a wide range of approaches (e.g., genetics and genomics, 3D quantitative imaging, growth modeling) to better integrate knowledge on reproductive development and apomixis; and a spectrum of scientific questions covering all aspects. Through research, training, and dissemination actions, the project will clarify the genetic architecture of apomixis and support the deployment of innovative strategies in crop improvement.

### 2.2. Work Packages Overview

The project proposes an interconnected portfolio of six work packages (WP) to ensure transparent and effective governance, compliance with scientific and education objectives, and wide visibility of objectives and achievements. WPs 1 and 2 are dedicated to Management & Coordination and to Communication & Dissemination, while each of the four remaining WPs addresses a unique scientific question using comparative and translational approaches. Any progress in a given WP is prone to fuel advances in the other ones.

In the first scientific WP (WP3), we will investigate the functional role of the heterochromatic genomic regions specific to apomicts genomes. To address this, we will decipher the structural and functional features of the locus controlling apomictic reproduction (Apomixis Controlling Locus, ACL) in natural apomictic grasses belonging to the Eragrostis and Paspalum genera (Figure 1A,B) and provide a general view of how apomictic genomes work as a whole. The proposed tasks will intend to unveil the gene content of the ACLs in several species under study and to compare its conservation, to identify regions prone to contain genes controlling key components of apomictic reproduction, and to propose hypotheses for the evolution of apomictic genomes.

The following WP (WP4) aims at characterizing genes regulatory mechanisms involved in apomixis control. Are the transcriptional patterns promoting apomixis shaped transcriptionally or post-transcriptionally? To address this question, WP4 is designed to typify the function of selected candidate genes for apomixis control, that belong to either transcriptional or post-transcriptional regulatory pathways, and to analyze the occurrence of changes in the epigenome of the respective mutants/transformants.

Next, in WP5, we will address the regulatory role of phytohormones, in particular, how do phytohormones interact to establish and control the reproductive cell fate during plant reproduction. To address this, we will two main questions: (a) how do auxin and cytokinin signaling coordinate MMC cell fate and female gametophyte development in sexual vs. apomictic Paspalum notatum, and (b) which of the differentially expressed relevant genes identified in the course of WP4 has a hormonal-related role in the specification of sexual vs. apomictic germline precursors cell fate? 

Finally, in WP6, we will ask whether ovule architecture also contributes to the control of apomixis and, notably, to female germline fate. To answer this question, this work package will build on preliminary results in Arabidopsis suggesting that altering ovule architecture changes the plasticity of reproductive cells fate in the ovule. By combining imaging and functional approaches, this WP focuses on the cellular parameters of the early ovule associated with, and predicting, the plasticity of the differentiation of the germline precursor cells, in sexual and apomictic plants.

### 2.3. Partner Institutions and Contact Persons

The MAD project is coordinated by Dr. Olivier Leblanc (PI) and Maïa Lejbowicz (project manager) from the Institut de Recherche pour le Développement (IRD), Plant Diversity Adaptation and Development Joint Research Unit (DIADE Univ Montpellier—CIRAD—IRD), France. In addition to IRD, participating institutions and contact persons include: University of Milan, Biosciences Department, Italy (Prof. Lucia Colombo); University of Perugia, Department of Agricultural, Food and Environmental Sciences, Italy (Prof. Emidio Albertini); University of Padova, Department of Agronomy Food Natural Resources Animals and Environment, Italy (Prof. Gianni Barcaccia); Institute of Biosciences and BioResources, National Research Council, Italy (Dr. Fulvio Pupilli); National Institute for Agricultural Biology (NIAB), Crop Bioinformatics group, UK (Dr. Mario Caccamo); University of Zürich, Department of Plant and Microbial Biology, Switzerland (Prof. Ueli Grossniklaus); CONICET—Universidad de Nacional de Rosario, Instituto de Investigaciones en Ciencias Agrarias de Rosario (IICAR), Argentina. (Prof. Silvina Pessino); CONICET—Universidad Nacional del Noreste, Instituto de Botánica del Nordeste (IBONE), Argentina (Dr. Francisco Espinoza); CONICET—Universidad Nacional del Sur, Centro de Recursos Naturales Renovables de la Zona Semiárida (CERZOS), Argentina (Prof. Viviana Echenique); University of Adelaide, School of Agriculture, Food and Wine, Australia (Prof. Matthew Tucker), and; CINVESTAV, Laboratorio Nacional de Genómica para la Biodiversidad (LANGEBIO), Mexico (Prof. Stewart Gillmor).

## 3. Abstracts of Presentations of the 7th Series of Seminars on Advances in Apomixis Research

Presentations focused on various issues pertaining to the biology of apomixis and apomictic forage grass breeding and were assembled into three sessions. The first one, Ovule development and reproductive fate, contains works investigating the control of female germline fate and how regulatory mechanisms are coordinated in both the sexual Arabidopsis model plant and apomictic grasses. The following section, Genomics and epigenomics of apomixis, addresses comparative genomics and epigenomics using different apomictic biological systems, to investigate the role of hybridity and polyploidy and examine the origin and nature of allelic variation at candidate loci. The last session, Agronomic traits and breeding approaches in apomictic forage grasses, focuses on aspects of germplasm enhancement and breeding in subtropical grass forages.

### 3.1. Ovule Development and Reproductive Fate

#### 3.1.1. Cloning of Diplosporous Apomixis Candidate Genes for Its Functional Characterization in the Model Plant *Arabidopsis thaliana*

Pasten MC *, Bellido A *, Garbus I and Echenique V

Centro de Recursos Naturales Renovables de la Zona Semiárida (CERZOS- CONICET) and Departamento de Agronomía, Universidad Nacional del Sur (UNS), Bahía Blanca, Argentina. * These authors contributed equally to this work: mcpasten@cerzos-conicet.gob.ar, andresbellido@gmail.com.

*Eragrostis curvula* is a perennial forage grass naturalized in semiarid regions of Argentina, with genotypes that can be either sexual diploids or polyploids (4×–8×) reproducing by diplosporous apomixis. Using different approaches, our research group obtained a list of candidate genes related to this reproductive mode that remain to be functionally characterized. The aim of this work is to optimize the construction of vectors for the introduction of these Eragrostis candidate genes into the model plant *Arabidopsis thaliana* in order to analyze their effects during reproductive development. Two strategies were used, Gateway and GoldenBraid, and vectors with single and multiple genes were introduced in *A. thaliana* plants using Agrobacterium and the floral dip method. In relation to GoldenBraid, focusing on the current knowledge about the complex dynamics that regulate germline specification in the ovule of *A. thaliana*, different promoters were selected, related to these key pathways, which will guide ectopic expression into the regions surrounding the MMC. Currently, using these promoters in the GoldenBraid system, constructs for each gene were generated, and vectors containing different combinations of genes were successfully created, using kanamycin or hygromycin for the selection of the transgenic plants. The phenotypic effects of these genes will be analyzed in the near future, using whole mount clearing and DIC microscopy.

#### 3.1.2. Early Ovule Morphogenesis and Germ Cells Formation

Autran D ^1,^*, Baroux C ^2^, Hernandez-Lagana E ^1^, Mosca G ^2^, Mendocilla Sato E ^2^, Pires N ^2^, Frey A ^2^, Giraldo-Fonseca A ^2^, Grossniklaus U ^2^, Hamant O ^3^, Godin C ^3^, Boudaoud A ^3,4^, Grimanelli G ^1^, Delgado L ^5^, Conejero G ^6^, Lartaud M ^6^

^1^ DIADE, University of Montpellier, CIRAD, IRD, Montpellier, France; ^2^ Department of Plant and Microbial Biology and Zurich-Basel Plant Science Center, University of Zürich, Zürich, Switzerland; ^3^ RDP, University of Lyon, ENS Lyon, UCB Lyon 1, CNRS, INRAE, INRIA, Lyon, France; ^4^ Present address: LadHyX, Ecole polytechnique, CNRS, Institut Polytechnique de Paris, Palaiseau, France; ^5^ Instituto de Investigaciones en Ciencias Agrarias de Rosario (IICAR), CONICET, FCA UNR, Rosario, Argentina. ^6^ AGAP, University of Montpellier, CIRAD, Montpellier, France. * daphne.autran@ird.fr

In flowering sexual plants, the female germline precursor differentiates as a single spore mother cell, so-called Megaspore Mother Cell (MMC). MMC formation is contemporary to early ovule primordium growth. Here, we explored how organ growth contributes to MMC differentiation. In the model plant Arabidopsis, by quantitative analysis of 3D images at cellular resolution, cell cycle markers, and tissue growth models, we identified spatio-temporal patterns of cell growth and divisions in the early ovule primordium. Our analysis revealed that MMC characteristics first arise in more than one cell, but MMC fate becomes progressively restricted to a single cell during organ growth. Altered primordium growth patterns in *katanin* mutants—affected in microtubules dynamics—coincided with a delay in the MMC fate restriction process. We propose a model of gradual canalization of reproductive cell fate coupled to ovule primordium growth in Arabidopsis. Further studies should determine if this model is conserved in ovules of grasses; either sexual such as in maize, or following an aposporous reproductive mode, such as in Paspalum.

#### 3.1.3. The RdDM Pathway Is Important to Specify a Single Female Germ Cell Precursor through the Restriction of Sporocyteless/Nozzle Expression

Mendes MA ^1,^*, Petrella R ^1^, Cucinotta M ^1^, Vignati E ^1^, Gatti S ^1^, Pinto SC ^2^, Bird DC ^3^, Gregis V ^1^, Dickinson H ^4^, Tucker MR ^3^, Colombo L ^1^

^1^ Dipartimento di Bioscienze, Università degli Studi di Milano, Via Celoria 26, 20133 Milano, Italy; ^2^ LAQV REQUIMTE, Departamento de Biologia, Faculdade de Ciências, Universidade do Porto, Rua do Campo Alegre s/n, 4169-007 Porto, Portugal; ^3^ School of Agriculture, Food, and Wine, The University of Adelaide, Waite Campus, Urrbrae, SA5064, Australia; ^4^ Department of Plant Sciences, University of Oxford, South Parks Road, Oxford OX1 3RB, UK. * marta.miranda@unimi.it

The female gametophyte formation starts from the differentiation of a single cell in the pre-meiotic ovule, named megaspore mother cell (MMC). Formerly, it was reported that mutants in RNA-dependent DNA methylation—RdDM pathway showed more than one MMC-like cell. We have recently shown that the DRM methyltransferase double mutant *drm1drm2* also presents supernumerary MMC-like cells, consistent with RdDM mutants’ phenotype. We have also demonstrated that SPOROCYTELESS/NOZZLE (SPL/NZZ) a transcription factor required for MMC specification is ectopically expressed in *ago9* and *drm1drm2* mutants suggesting that the multiple MMC-like phenotype might be due to its ectopic expression. Interestingly we show that the ovule identity gene SEEDSTICK, directly regulates the two RdDM players *AGO9* and *RDR6* expression in the ovule and, therefore, indirectly SPL/NZZ. Based on our and previous results, we have so proposed a model describing the network required to restrict SPL/NZZ expression to specify a single MMC. In the future, our focus will be to uncover in more detail the mechanisms controlling MMC specification and, in particular, *SPL/NZZ* expression.

#### 3.1.4. Depletion of Trimethylguanosine Synthase1 Alters Reproductive Development in *Arabidopsis*

Siena LA ^1^,*, Michaud C ^2^, Selles B ^2,3^, Pessino SC ^1^, Ingouff M ^2^, Ortiz JPA ^1^, Leblanc O ^2^

^1^ Instituto de Investigaciones en Ciencias Agrarias de Rosario (IICAR), CONICET, FCA UNR, Rosario, Argentina; ^2^ DIADE, Univ Montpellier, CIRAD, IRD, Montpellier, France; ^3^ Current address: Université de Lorraine, INRAE, IAM, F-54000 Nancy, France. * siena@iicar-conicet.gob.ar

In flowering plants, apomixis refers to asexual reproduction through seeds. Recently, we identified a plant ortholog of yeast *trimethylguanosine synthase1* (*TGS1*), whose expression is positively correlated with the rate of sexuality in reproductive tissues of facultative apomictic *Paspalum notatum* genotypes. In yeast, mammals and insects, TGS1 is encoded by a single gene and performs multiples activities. It catalyzes 5′ cap trimethylation of noncoding RNAs, plays a role in the biogenesis of sn(o)RNAs, rRNA processing, and telomerase function. Moreover, it acts as a transcriptional co-activator associated with PRIP. The structure and function of *TGS1* orthologs in plants remain poorly characterized. Plants contain a specific copy with a WW interaction domain at the N-terminal. To explore *TGS1* possible association with apomixis, we determined its expression patterns in Arabidopsis and studied its loss of function in two T-DNA insertion lines. RT-PCR revealed preferential expression of *TGS1* in reproductive tissues. Transgenic plants carrying synthetic reporter constructs (*pTGS1*:*TGS1*: GUS/GFP) showed it is expressed throughout female gametogenesis and embryogenesis, but not in the nucellus. Mutant plants showed reduced fertility, and mutant allele transmission suggested gametophytic effects, with stronger bias when maternally transmitted. Cytoembryological analyses showed apomixis-like phenotypes, including multiple megaspore mother cells and embryo sacs, and extra embryos. Cell-identity markers revealed cell-fate alterations during both female gametogenesis and early seed development. Moreover, germination rate and seedling defects were observed. The knowledge gained indicates an essential role for *TGS1* during sexual female gametogenesis and embryo patterning in plants.

#### 3.1.5. Preliminary Studies of the Role of TGS1 in sRNA Methylation and Splicing during Paspalum Notatum Reproductive Development

Colono CM *, Podio M, Pessino SC

Instituto de Investigaciones en Ciencias Agrarias de Rosario (IICAR), CONICET, FCA UNR, Rosario, Argentina. * colono@iicar-conicet.gob.ar

*Paspalum notatum* is one of the main apomixis grass models. Previous transcriptome analysis further validated by mRNA in situ hybridization revealed that the gene *TGS1* (encoding the RNA methyltransferase *TRIMETILGUANOSINE SYNTHASE 1*) is repressed in ovules of apomictic *P. notatum* plants. In animals and/or yeasts, TGS1 promotes the processing several RNA types and also acts as a transcriptional co-activator. Recently, we reported that silencing of *TGS1* causes the emergence of foliar trichomes and supernumerary aposporous-like embryo sacs in *P. notatum*, which led to its classification as a developmental repressor. Our objective here was to analyze whether *TGS1* influences RNA processing in this species. Three-hundred and sixteen (316) RNA transcripts differentially expressed in flowers of apomictic and sexual plants were selected to examine the existence of putative splice variants. Using qRT-PCR analysis, we confirmed that an unprocessed splicing variant of one of them (*CHLOROPHYLL A-B BINDING PROTEIN 1B-21*, renamed *CHLORO*) is less represented in apomictic plants at a statistically significant level. The same unprocessed splicing variant also diminished in antisense *tgs1* sexual lines. Moreover, since we identified seven miRNAs upregulated in floral sRNA libraries of sexual *P. notatum* plants, we are testing their representations in the same *tgs1* antisense lines. Our conclusion is that *TGS1* influences the splicing of CHLORO, a component of the photosynthesis light-harvesting the photosynthesis antenna, which might relate with the role of this gene as leaf trichomes formation repressor. The function of TGS1 in miRNA regulation remains unclear.

#### 3.1.6. Identification of *PPI_FKBP* Transcripts Expressed during *Paspalum notatum* Reproductive Development and Their Relationship with Apomixis

Stein J *, Spoto N, Podio M, Ortiz JPA

Instituto de Investigaciones en Ciencias Agrarias de Rosario (IICAR-CONICET-UNR), Zavalla, Argentina. * jstein@unr.edu.ar

In *Paspalum notatum* apomixis is governed by a single dominant 36 Mpb-long superlocus (ACL), showing strong restriction of recombination and association with a highly methylated heterochromatic knob. Genomic sequencing revealed the presence of repetitive segments and single-copy genes, while synteny studies indicated its location into a ‘hybrid’ genomic region carrying markers from rice chromosomes 2 and 12. Particularly, the RFLP marker C932 (rice Chr. 2), which encodes for a FKBP-type peptidyl-prolyl isomerase (*PPI_FKBP;* Os02g0760300), strictly cosegregates with apomixis. Our objective here was to start characterizing the expression of the ACL-derived *PPI_FKBP*, to enable further reproductive functional analysis. Eight (8) transcripts showing homology to C932 were retrieved from reference reproductive transcriptomes of the species. Five (5) of them, all derived from the same gene (isogroup) and encoding functional proteins, were detected in sexual genotypes. Other 3, originated from different genes (isogroups) and displaying dissimilar structures, were detected in apomictic transcriptomes. One of the apomictic sequences encodes for a functional protein, another one derives from a pseudogene, and the last one is a chimeric transcript, with homology to *PPI_FKBP* and *DNAJ* (the adjacent gene in the rice genome) (PPIQUIM). In experimental mapping, only PPIQUIM resulted genetically linked to the ACL. However, genomic sequencing indicated that *PPI_FKBP-* and *DNAJ*-derived exons are separated by a long intergenic region. Moreover, PPIQUIM was exclusively amplified from floral RNA of apomictic plants. Our results indicate that PPIQUIM is specifically expressed from the ACL and would be a product of intersplicing. Its functional role remains to be determined.

#### 3.1.7. Disclosing the Molecular Link between Apospory and Diplospory

Garbus I ^1^, Podio M ^2^, Echenique V ^1^, Pessino SC ^2,^*

^1^ Centro de Recursos Naturales de la Zona Semiárida (CERZOS-CONICET-UNS), Bahía Blanca Argentina; ^2^ Instituto de Investigaciones en Ciencias Agrarias de Rosario (IICAR-CONICET-UNR), Zavalla, Argentina. * pessino@iicar-conicet.gob.ar

Gametophytic apomixis forms seeds with clonal embryos identical to the mother plant through two different mechanisms, namely apospory and diplospory, both of them involving the formation of non-reduced megagametophytes within the ovule. These non-reduced embryo sacs are originated from companion nucellar cells (apospory) or the megaspore mother cell, after absence/failure of meiosis (diplospory). In both paths the non-reduced egg cells produce embryos through parthenogenesis. Our hypothesis here was that, at least for some candidate genes, apomeiosis and parthenogenesis associate, respectively, with contrasting or identical representation patterns in aposporous vs. diplosporous differential mRNA expression comparisons. The rationale supporting this proposition is that in aposporous and diplosporous plants apomeiosis might involve some common genes under an opposite spatio-temporal regulation pattern (OE transcripts), while parthenogenesis should be identically activated in both reproductive types (IE candidates). We compared two sets of Roche/454 sequences differentially expressed in reproductive organs of sexual vs. apomictic plants of *Paspalum notatum* (aposporous) and *Eragrostis curvula* (diplosporous) from pre-meiosis to anthesis, to identify OE and IE transcripts. Then, the selected sequences were investigated in TruSeq/Hiseq Illumina RNA floral libraries constructed at different reproductive stages. We found 89 opposite expression transcripts exclusively represented at pre-meiosis and 66 equal expression transcripts occurring at anthesis only. These candidate genes were used to produce String interaction networks with others previously related with apomeiosis and parthenogenesis, and their location is being mapped within the *Paspalum* and *Eragrostis* genomic ACL (apomixis controlling loci). Functional analysis of selected sequences is at work.

#### 3.1.8. Genes Modulating the Increase of Sexuality in the Facultative Apomictic Grass Eragrostis Curvula under Water Stress Conditions

Selva JP *, Zappacosta D, Carballo J, Rodrigo JM, Bellido A, Gallo CA, Gallardo J, Echenique V

Centro de Recursos Naturales de la Zona Semiárida (CERZOS-CONICET-UNS), Bahía Blanca Argentina. * jpselva@cerzos-conicet.gob.ar

Previous reports in *Eragrostis curvula*, a diplosporous apomictic forage grass, showed that facultative apomictic genotypes reduce the proportion of apomictic embryo sacs under stressful conditions like in vitro culture and drought. Here, we report the cytoembryological and transcriptomic analysis of inflorescences of facultative apomictic plants under control and drought stress treatment in order to identify the mechanisms regulating the switch from sexual to asexual reproduction. Embryo sacs from control and stressed plants (161 and 172, respectively) were analyzed, and RNA-Seq Illumina libraries with three biological replicates from inflorescences of control and stressed plants were sequenced. The percentage of sexual embryo sacs increased from 4 to 24% and 501 out of 201,011 transcripts were differentially expressed (DE) between control and stressed plants (151 upregulated and 350 downregulated in stressed plants). Out of these, 380 were annotated and analyzed using BLAST2GO and KEGG. DE transcripts were compared with previous transcriptomes where apomictic and sexual genotypes were contrasted. The results point as candidates to transcripts related to methylation, ubiquitination, hormone and signal transduction pathways, transcription regulation, and cell wall biosynthesis, some acting as a general response to stress, and some that are specific to the reproductive mode. We suggest that a DNA glycosylase EcROS1-like could be demethylating, thus de-repressing a gene or genes involved in the sexual pathway. Many of the other DE transcripts could be part of a complex mechanism that regulates apomixis and sexuality in this grass, the ones in the intersection between control/stress and apo/sex being the strongest candidates.

### 3.2. Genomics and Epigenomics of Apomixis

#### 3.2.1. Mapping Apomixis in the Plicatula Group of Paspalum through Genotyping by Sequencing

Wagner AW ^1,^*, Ortiz JPA ^2^, Espinoza F ^1^

^1^ Instituto de Botánica del Nordeste (IBONE), CONICET, FCA UNNE, Corrientes, Argentina; ^2^ Instituto de Investigaciones en Ciencias Agrarias de Rosario (IICAR-CONICET-UNR), Zavalla, Argentina. * werfilwagner@gmail.com

In the *Paspalum*, apomixis (asexual reproduction via seeds) is controlled by a single-dominant complex locus (ACL). The *Plicatula* group includes species either with diploid sexual (2n = 2x = 20) and apomictic tetraploid (2n = 4x = 40) cytotypes, and with exclusively polyploid (mostly tetraploid) races. While genetic linkage maps in *P. notatum* and *P. simplex* revealed non-recombinant ACLs, initial AFLP mapping in *P. guenoarum* (Plicatula) revealed a recombinant ACL. This work aims at building a dense genetic linkage map of tetraploid *Plicatula* spp. through genotyping by sequencing (GBS), localizing the ACL and facilitating both identification and positional cloning of the genes governing apomixis. The mapping population (n = 82; 52 sexual and 32 apomict F_1_ plants) derives from a cross between *P. plicatulum* 4-PT (a 4× colchicine-induced sexual plant) × *P. guenoarum* GR19 (a 4× natural apomict individual). Genomic DNA was digested with *ApeK*I and used for preparing Illumina NextSeq 550 libraries. A total of 366 million reads (75 bp single-end; 4.35 million reads/sample on average) were obtained. Initially, the STACKS and TASSEL_UNEAK softwares were used for *de novo* SNP discovery, but failed to identify relevant markers. Then, the TASSEL5GBSV2 pipeline was used with *Setaria viridis*, *Zea mays,* and *P. notatum* (draft) genomes as references and produced, respectively, 9,264; 2,365, and; 10,711 single-dose markers. Genotypes related with the ACL (Aaaa and aaaa for apomictic and sexual plants, assuming a dominant simplex control) were assigned to each F1 in the GBS data matrix. Linkage analyses are ongoing using JoinMap 4.0 and a pseudo-test cross model.

#### 3.2.2. Sequencing Paspalum Genomes

Leblanc O ^1,^*, Pupilli F ^2^, Albertini E ^3^, Espinoza F ^4^, Pessino, SC ^5^, Ortiz JPA ^5^

^1^ DIADE, Univ Montpellier, CIRAD, IRD, Montpellier, France; ^2^ Institute of Biosciences and Bioresources, Research Division of Perugia, National Research Council, Perugia, Italy; ^3^ Department of Agricultural, Food and Environmental Science, University of Perugia, Perugia, Italy; ^4^ Instituto de Botánica del Nordeste (IBONE), CONICET, FCA UNNE, Corrientes, Argentina; ^5^ Instituto de Investigaciones en Ciencias Agrarias de Rosario (IICAR-CONICET-UNR), Zavalla, Argentina. * olivier.leblanc@ird.fr

*Paspalum notatum* is a forage grass from South American subtropical regions. Diploid individuals (2n = 20) are sexual plants, while polyploid individuals reproduce through aposporous apomixis. In grasses, apomixis is genetically controlled by a single non-recombinant heterochromatic genomic region, the Apomixis Controlling Region (ACR) which nature and function remain elusive. Our objective is to generate genomic sequences of sexual and apomictic *P. notatum* individuals to understand ACR emergence and evolution; to identify genes governing important agronomical traits, including apomixis; and to improve genomic selection in breeding programs. To achieve this, we selected one diploid genotype (R1) and two tetraploid genotypes reproducing through obligate (Q4188) and facultative (Q3664) apomixis. Genomic DNA was sequenced using Illumina (R1) and Oxford Nanopore (R1, 32 Gb; Q4188, 22 Gb; Q3664, 21 Gb) technologies. K-mer analyses using short reads confirmed flow cytometrical analyses for R1 genome size (~600 Mbp) and high heterogozigosity. Here we report on the quality metrics of R1 genomic assemblies generated by combining the output of several long-read assemblers (wtdbg2, Shasta, Flye, miniasm, and smartdenovo) and Bionano optical maps: size, N50, contiguity, BUSCO completeness, coverage distribution. The best hybrid scaffolding using a 2300-contig smartdenovo assembly rendered 53 scaffolds (795.27 Mbp, N50 33.54 Mbp, >95% BUSCO completeness). However, a partial representation of heterozygous regions and numerous, dispersed gaps still hinder structural variation analyses. We anticipate that a new set of ONT sequences and the use of tools for detecting and purging haplotypes will allow in a short time the completion of the assembly of R1 genome haplotypes.

#### 3.2.3. Identification of a Genomic Region Linked to Apomixis in Eragrostis Curvula

Carballo J ^1,^*, Gallardo J ^1,^*, Gallo CA ^1^, Selva JP ^1^, Garbus I ^1^, Zappacosta D ^1^, Albertini E ^2^, Caccamo M ^3^, Echenique V ^1^

^1^ Centro de Recursos Naturales de la Zona Semiárida (CERZOS-CONICET-UNS), Bahía Blanca Argentina; ^2^ Department of Agricultural, Food and Environmental Science, University of Perugia, Perugia, Italy; ^3^ NIAB, Cambridge, Reino Unido. * These authors contributed equally to this work: jcarballo@cerzos-conicet.gob.ar, jgallardo@cerzos-conicet.gob.ar

*Eragrostis curvula* is a forage grass with n = 10 and ploidy levels ranging from 2× to 8×. This grass has become a model for the study of diplosporous apomixis, an asexual reproduction by seeds in which the progeny is genetically identical to the mother plant. To identify the genomic regions and the genes involved in apomixis, the genome of the diploid sexual cultivar Victoria was sequenced using a combination of approaches (PacBio, Chicago and Hi-C) and contrasted against the region identified by high density mapping. The final assembly render an N50 of ~43 Mb and 1143 contigs being completely assembled the seven longest full chromosomes according to the synteny analyses. The annotation identified 56,469 genes and 28.7% of repetitive elements. On the other hand, a high saturated linkage map at tetraploid level was constructed using both traditional (AFLP and SSR) and high-throughput molecular markers (GBS-SNP) and a locus controlling diplospory and putative regulatory regions affecting the expressivity of the trait were identified. The four markers linked to apomixis in the *E. curvula* linkage map were aligned to Victoria, identifying a 10 Mb region. Furthermore, using a transcriptomic approach, genes up and downregulated in the apomictic genotypes were identified, including genes that could be repressing sexuality or promoting apomixis. The identification of the region linked to apomixis, its expression and regulation could allow to better understand some features of this interesting trait and it paves the way to sequence and assemble the complete region in the more complex polyploid genotypes.

#### 3.2.4. A Deeper Understanding of Differential Sense/Antisense RNA Representation in Reproductive Organs of Sexual and Apomictic Paspalum Notatum

Podio M *, Colono C *, Siena L, Ortiz JPA, Pessino SC

Instituto de Investigaciones en Ciencias Agrarias de Rosario (IICAR-CONICET-UNR), Facultad de Ciencias Agrarias, Universidad Nacional de Rosario, Campo Experimental Villarino, Zavalla, Santa Fe, Argentina. * These authors contributed equally to this work: podio@iicar-conicet.gob.ar, colono@iicar-conicet.gob.ar

The selection of apomixis genes to be used as breeding/research tools requires detailed characterization of the expression landscape across multiple developmental stages. Our group established Roche 454/FLX+ reference floral transcriptomes from sexual and apomictic *Paspalum notatum* biotypes and used these solid frames to assist the assembly of 24 extensive TruSeq^®^/Hiseq Illumina libraries, covering in triplicate four reproductive steps (pre-meiosis, meiosis, post-meiosis, anthesis). Moreover, *de novo* assemblies were constructed with Trinity to complete an integrative global transcriptome. For differential expression analysis, RNA-seq reads were analyzed with the Kallisto v.0.44.0 software to determine transcript counts and abundances. These analysis allowed us to: (1) identify sequences differentially expressed in apomictic and sexual biotypes, which were consistently differential on the course of development or otherwise stage-specific; (2) characterize some differentially expressed transcript clusters (particularly transcription factors of the AP2, ARF, MYB, and WRKY groups, as well as hormone-pathways controllers of the auxin, jasmonate, and cytokinin families); (3) detect a number of antisense sequences differentially modulated between reproductive modes; and (4) gain knowledge on the expression of a group of transcripts that had been previously associated with apomixis. Currently, we are using the Illumina libraries to identify transcripts expressed from the apomixis controlling locus (ACL), whose sequence is partially available through the *Paspalum* genome sequencing initiative.

#### 3.2.5. Is Apomictic Seed Development Influenced/Controlled by Epigenetic Factors?

Marconi G ^1^, Soliman M ^2,3^, ZAappacosta D ^4^, Di Marsico M ^1^, Bocchini M ^1^, Gallardo J ^4^, Delgado L ^2,3^, Echenique V ^4^, Albertini E ^1,^*

^1^ Department of Agricultural, Food and Environmental Sciences, University of Perugia, Perugia, (Italy); ^2^ Instituto de Investigaciones en Ciencias Agrarias de Rosario (IICAR-CONICET-UNR), Zavalla, Argentina; ^3^ Facultad de Ciencias Agrarias, Universidad Nacional de Rosario, Zavalla (Argentina); ^4^ Centro de Recursos Naturales de la Zona Semiárida (CERZOS-CONICET-UNS), Bahía Blanca, Argentina. * emidio.albertini@unipg.it

Several lines of evidence suggest that transitions during reproduction and early seed development are epigenetically regulated by dynamic changes in chromatin state. Moreover, deregulation of key developmental steps in sexual processes are thought to cause apomixis, and supporters of this hypothesis justify it based on the coexistence of sex and apomixis in the same individual. For example, some authors found that DNA methylation deregulation in reproductive cells induce apomeiosis-like phenotypes suggesting that specialization of a DNA methylation pathway acts upon germline or germline associated cells. With this aim in mind, we decided to investigate DNA methylation differences between apomictic and sexual genotypes of two species, *Paspalum rufum* and *Eragrostis curvula*. High-throughput DNA sequencing technologies have enabled the measurement of cytosine methylation on a genome-wide scale. Many technologies have been developed over the past decade to measure DNA methylation. MCSeEd (Methylation Content Sensitive Enzyme ddRAD) is a reduced-representation, reference-free, cost-effective approach for characterizing whole genome methylation patterns across different methylation contexts (CG, CHG, CHH, 6 mA) that we have recently developed at the University of Perugia. DNA from triplicate panicle samples of contrasting reproductive mode materials were digested with enzymes sensitive to DNA methylation (*Aci*I, *Pst*I, *Eco*T22I, and *Dpn*II, for the CG, CHG, CHH and 6 mA contexts, respectively), and libraries were generated and sequenced in Illumina platform. Several differentially methylated genomic regions were found and associated to annotated genes that were classified with the BLAST2GO software. Several genes already linked to apomixis (i.e., SERK, APOSTART) were found to be both differentially methylated and differentially expressed.

#### 3.2.6. Epigenetic Marks Associated with Apospory Expressivity during Floral Development of Diploid Paspalum Rufum Cytotype

Delgado L ^1,^*, Soliman M ^1^, Podio M ^1^, Marconi G ^2^, Di Marsico M ^2^, Ortiz JPA ^1^, Albertini E ^2^

^1^ Instituto de Investigaciones en Ciencias Agrarias de Rosario (IICAR-CONICET-UNR), Zavalla, Argentina; ^2^ Department of Agricultural, Food and Environmental Sciences University of Perugia, Perugia, Italy. * luciana.delgado@conicet.gov.ar

Apomixis is an asexual reproduction pathway through seeds that generates clonal progenies. It was proposed to derive from the deregulation of genes involved in sexuality by genetic/epigenetic mechanisms. *Paspalum rufum* grass forms a multiploid complex with self-sterile sexual diploid and self-fertile apomictic tetraploid cytotypes but some diploid genotypes produce aposporous embryo sacs and clonal seeds. Moreover, variation in apospory expressivity was detected within experimentally obtained diploid families. Here we compare the relative methylation levels between floral libraries of full-sib diploid *P. rufum* genotypes exhibiting low and high apospory expressivity. Methylation Content Sensitive Enzyme ddRAD (or MCSeEd) strategy, without reference genome, was applied in two developmental stages, pre-meiosis/meiosis and post-meiosis and at three different contexts (CG, CHG and CHH). Heatmaps and principal component analysis of the relative methylation changes clearly discriminated between reproductive behavior at both developmental stages and for almost all the methylation contexts. *P. notatum* floral transcriptome was queried by differential methylated contigs (DMCs), revealing 14% of homology, almost half of which were differentially expressed between apomictic and sexual samples of *P. notatum*. BLAST searches over public databases allowed the identifications of DMCs homologous to genes involved in flower growth, development and also to many genes and genomic regions previously associated to apomictic development. In silico mapping on *Setaria italica* genome showed a high proportion of DMCs aligning on genomic regions previously associated with apomixis. This work provides evidence of the presence of differential methylation levels associated with variation in apospory expressivity in a diploid grass species.

#### 3.2.7. Epigenetic Mechanisms Involved in Diplospory Regulation in Weeping Lovegrass

Zappacosta DC ^1,2,^*, Garbus I ^1,3,^*, Selva JP ^1^, Carballo J ^1,2^, Pasten MC ^1^, Gallardo JA ^1,2^, Bellido AM ^1^, Marconi G ^1,4^, Albertini E ^1,4^, Echenique V ^1,3^

^1^ Centro de Recursos Naturales de la Zona Semiárida (CERZOS-CONICET-UNS), Bahía Blanca, Argentina; ^2^ Depto. Agronomía-UNS Bahía Blanca, Argentina; ^3^ Depto. Ciencias de la Salud-UNS Bahía Blanca, Argentina; ^4^ Università degli Studi di Perugia, Dip. di Scienze Agrarie, Alimentari e Ambientali, Perugia, Italy. * These authors contributed equally to this work: dczappa@criba.edu.ar, igarbus@criba.edu.ar

Weeping lovegrass (*Eragrostis curvula*) is a forage grass with a particular diplosporous apomixis where meiosis is absent and sexual and apomictic seeds have equal embryo:endosperm ploidy ratio. Several lines of evidence show that epigenetic regulation is involved in the expressiveness of the trait, accounting for the variable expression between plants and for the increase of the frequency of sexual pistils in facultative genotypes triggered by biotic and abiotic stresses. The aim of this study was to associate differences in the reproductive mode to epigenetic mechanisms given by DNA methylation and miRNAs regulation of gene expression. Firstly, the Methylation Content Sensitive Enzyme ddRAD (MCSeEd) was used to detect hypermethylated and hypomethylated positions and further associate them with apomictic (full and facultative) and sexual genotypes. The distribution of differentially methylated positions across genes at CG and 6mA shows a peak around the transcript start and transcript termination sites, hence regulating gene expression mainly through the incorporation of methyl groups on these positions. Gene ontology analysis reveals terms associated to auxins, clathrins, and kinases previously observed in apomictic species. Moreover, some genes differentially expressed related to the reproductive pathway, could be linked to the methylation status in the present work. Regarding miRNAs, a target analysis allowed the identification of a MADS-box transcription factor and a transposon repressed in the sexual genotype through specific miRNA-mRNA interactions. Additional miRNA-mRNA pairs specific for sexual or apomictic genotypes were identified in a degradome-based analysis conducted using *Oryza sativa* database, reinforcing the concept of apomixis resulting from sexual pathway deregulation.

### 3.3. Agronomic Traits and Breeding Approaches in Apomictic Forage Grasses

#### 3.3.1. Flowering Induction in *Paspalum plicatulum* Michx, a Sexual Tetraploid and Sexual Plant

Novo PE ^1,^*, Ruiz Díaz GS ^1^, Quarin CL ^1^, Vidoz ML ^1,2^, Espinoza F ^1,2^

^1^ Facultad de Ciencias Agrarias, Universidad Nacional del Nordeste (FCA-UNNE), Corrientes, Argentina; ^2^ Instituto de Botánica del Nordeste (IBONE-CONICET), Corrientes, Argentina. * patriciaenovo@gmail.com

*Paspalum plicatulum* is an experimental sexual autotetraploid plant (4 × S) obtained by colchicine doubling chromosomes from a wild 2× plant. 4 × S had already produced interspecific hybrids in crossing with tetraploid apomictic species (4 × A) of the Plicatula group. However, the 4 × S plant blooms in early summer and there are some 4 × A species in this group which bloom in late April, preventing crossbreeding. The objective of this study was to delay the flowering of 4 × S by controlling the photoperiod and temperature and to hybridize with 4 × A species of the Plicatula group that flower in April. The experiments were carried out in a chamber with controlled light (14 h) and temperature (25 °C ± 2°) and were repeated twice. We used 16 4 × S clones in a 4-treatment design: (1) control (in greenhouse, natural light), (2) light intensity of 170 μmol m^−2^ s^−1^ of; (3) light intensity of 350 μmol m^−2^ s^−1^; and (4) light intensity of170 μmol m^−2^ s^−1^ + 25 µg GA_3_ en 10 µL of ethanol 95% (*v*/*v*). The control did not flower, while all the chamber treatments started flowering at 90 days. Using these plants as pistillate, hybrids were obtained after crossing with cv. Cambá and line U44 of *P. atratum* and with cv. Chané of *P. guenoarum*. Thus, the delay in flowering of the 4 × S plant allowed obtaining new interspecific hybrids to be achieved within the Plicatula group of *Paspalum*. This will permit expanding the use of germplasm from other apomictic species in the genetic improvement program for native forages.

#### 3.3.2. Heterosis in Tetraploid *Paspalum notatum*: Evaluation of Its Occurrence, Prediction, and Breeding Techniques

Marcón F *, Martínez EJ, Brugnoli EA, Zilli AL, Acuña CA

Instituto de Botánica del Nordeste, CONICET, Facultad de Ciencias Agrarias, UNNE, Corrientes, Argentina. * fmarcon91@gmail.com

*Paspalum notatum* Flüggé is one of the main components of the South American grasslands. At present, hybridization is the most popular breeding technique in the species, and its goal is to obtain superior apomictic hybrids. Occurrence of heterosis in tetraploid *P. notatum* hybrid progenies in relationship with the genetic distance among parents was determined. Secondly, recurrent selection based on combining ability (RSCA) and recurrent phenotypic selection (RPS) were evaluated in a sexual synthetic tetraploid population (SSTP). Genetic distances among parents with different origins were determined using molecular markers. Group of crosses among parents with low, intermediate and high genetic distances were identified. The progeny obtained was evaluated for a series of agronomic and morphological traits. There was a significant relationship between genetic distances among parents and heterosis mainly for forage yield. For this reason, molecular markers could be used as a tool to predict the occurrence of heterosis for this trait. On the other hand, two new sexual populations were created by RSCA and RPS. Sexual genotypes obtained by both methods were crossed with superior apomictic genotypes. Both methods allowed us to obtain hybrid progenies that were evaluated for summer, fall, and spring growth. RPS progenies exhibited greater summer growth and heterosis than RSCA progenies, although for fall and spring growth were similar. RPS was equal or more efficient than RSCA since it allowed obtaining equal or more genetic progress and heterosis. The breeding techniques used in this work allow exploiting the heterosis in tetraploid *P. notatum*.

#### 3.3.3. Advances in the Breeding of Paspalum Notatum towards a New Forage Cultivar

Acuña CA *, Zilli AL, Brugnoli EA, Marcón F

Instituto de Botánica del Nordeste, CONICET, Facultad de Ciencias Agrarias, UNNE, Corrientes, Argentina. * caalac77@gmail.com

There is a need for developing forage cultivars for subtropical areas, which combine warm summers with cold winters, and marked variation in photoperiod. *Paspalum notatum* is an apomictic grass adapted to these transitional climate zones. The objective was to describe the advances of the *Paspalum notatum* breeding program in Argentina. Crosses were made between a few sexual clones and several apomictic ecotypes collected throughout the New World. Segregation for mode of reproduction varied between 1:1 to 1:7.4 between apomictic and sexual within families, with a mean of 1:3.2. A high variation was observed for apomixis expressivity within the apomictic progeny, with most hybrids exhibiting very low (less than 10% of the ovules bearing aposporous embryo sacs) or high expressivity (more than 70%). Only the highly apomictic hybrids were evaluated in the field as spaced plants for two years and sward plots for three years, especially considering frost tolerance, and growth during the cool season. Currently, five selected hybrids are being evaluated in three locations in northeastern, and one in central Argentina. A grazing trial is also being conducted to determine the grazing tolerance of these 5 hybrids. The genetic stability across cropping cycles will also be determine using molecular markers. A superior highly apomictic genotype is expected to be selected in two years.

#### 3.3.4. Review of the Breeding Process of Apomictic Hybrids in Forage Grasses at the Research Center for Tropical Agriculture—CIAT

Castiblanco V *

International Center for Tropical Agriculture (CIAT), Km 17, Recta Cali-Palmira, Palmira, Valle del Cauca, Colombia. * v.castiblanco@cgiar.org

The forages breeding program at CIAT has a 45-year history, and it aims to improve the livelihoods of poor crop-livestock producers in the tropics, by intensifying production while reducing the environmental footprint. Furthermore, adaptation of forages to droughts and waterlogging related to global climate change is a priority to secure animal nutrition. The forage breeding program of CIAT aims to tackle this by developing hybrids with desired traits that are targeted for different environments. To date, our program works on three breeding pipelines: *Urochloa interspecific*, *Urochloa humidicola* and *Megathyrsus maximus* (formerly known as *Brachiaria interspecific*, *Brachiaria humidicola,,* and *Panicum maximus*). The *U. interspecific* breeding pipeline has developed interspecific hybrids for sub-humid tropics that are adapted to extreme biotic stress (e.g., spittlebug insects) and abiotic conditions (e.g., acid soils), with improved quality and maximum biomass production. This pipeline released in 2001, the first apomictic hybrid in the field of tropical forages and exhibits the most successful hybrid in the market currently: Mulato II. To date, six apomictic hybrids are available in the market. In addition, we are preparing products for the humid tropics (*U. humidicola*) and another for highly intensified livestock systems (*M. maximus*). We presented an overview of the genotyping tools (e.g., marker-assisted selection for apomixis), phenotyping tools (e.g., spittlebug screening), as well as the integration of these approaches into a recurrent selection scheme, enabling the permanent release of high-performing hybrids with constant genetic gain.

#### 3.3.5. Advances in the Breeding of *Panicum maximum* in Brazil

Jank LJ *, Santos, MF, Valle CB do, Carvalho SB, Simeão RM, Raposo A, Chiari L, Meirelles K

Embrapa Beef Cattle, Campo Grande, MS, Brazil. * liana.jank@embrapa.br

Breeding of *Panicum maximum* Jacq. (syn. *Megathyrsus maximus* (Jacq.) B.K.Simon & S.W.L.Jacobs) in Brazil began in 1982 with the introduction of the ORSTOM (Institut de Recherche pour le Développement) germplasm collected in East Africa and composed of 426 apomictic accessions and many sexual tetraploid plants. Field evaluation of the apomictic accessions in Campo Grande, MS, Brazil, led to the selection of 25 which were evaluated in seven regions. The best ones were evaluated under grazing and released as cv. Tanzânia. Mombaça and Massai in 1990, 1993, and 2001, respectively. Nowadays, they occupy around 20 million hectares in Brazil and respond for about 96% of the commercialized seeds of the species. Recently, other three cultivars were released. BRS Zuri is a tall wide-leaved high yielding high-quality cultivar, which resulted in 13% higher animal gain/area than Tanzânia and Mombaça in the Amazon and Cerrado biomes. BRS Tamani is a short, narrow-leaved plant which resulted in 10–16% higher gain/animal than Massai due to 6–20% higher crude protein and digestibility. BRS Quênia is medium-sized, medium-leaved, high yielding high-quality cultivar which resulted in 9% higher animal gains/area than Tanzânia in the Amazon biome and 17% higher than Mombaça in the Cerrado biome. Advances in genetic information were obtained from the evaluation of over 8000 hybrids from crosses between 10 divergent sexual plants and 10 divergent apomictic accessions on two levels of soil fertility. Advances in the program include high-resolution linkage map and genomic selection with allele dosage, large-scale phenotyping, and obtention of core collections.

#### 3.3.6. Reproductive Behavior of the Apomictic Paspalum Notatum Hybrid Cultivar Boyero-UNNE Growing at Field Conditions in the Argentina Temperate Region

Anibalini VA *, Busnelli V, Balaban D, Soliman M, Martín, B, Delgado L, Ortiz JPA

Instituto de Investigaciones en Ciencias Agrarias de Rosario (IICAR-CONICET-UNR), Zavalla, Argentina. * veronica.anibalini@unr.edu.ar

*Paspalum notatum* cv. Boyero-UNNE is an apomictic-hybrid cultivar developed at FCA-UNNE, Argentina. Here we evaluated seed development and phenotypic/reproductive stability through successive generations of Boyero-UNNE in the argentine temperate region (pampas). Plots were established at the FCA-UNR, Zavalla, Argentina. The development of seeds was examined by collecting 20 inflorescences every 3 days during the flowering period (21 days in total) and observing ≥50 spikelets/plant with a binocular microscope. Proportions of small fertilized ovaries (SFO), immature caryopses (IC), filled caryopses (FC), necrotic ovaries (NO), and empty spikelets (ES) were scored. The stability of vegetative and reproductive traits was evaluated in three F_1_ families (seeds collected in 2016, 2017, 2018) by measuring plant height (PH), tiller number (TN), stem length (SL), bunch length (BL) and percentages of ovules carrying aposporous sacs (% OAES), meiotic sacs (% OMES) and aposporous + meiotic sacs (% OMIX). The vegetative traits were also examined in the progeny of each F_1_ family. Seed development studies showed 18% SFO, 18% IC, 38% FC, 24% NO, and 2% ES. Non-significant differences were observed in vegetative traits across families and generations. The reproductive characterization showed, on average, 54.7% OAES, 15.4% OMES and 29.9% OMIX (total apomixis capacity: % OAES +% OMIX = 84.6%). Our results indicated that cv. Boyero-UNNE can form caryopses in up to 74% of the spikelets (18% SFO, 18% IC, and 38% FC). Moreover, considering that original Boyero-UNNE reports notify a total apomictic capacity of 86–93%, both vegetative and reproductive traits can be considered stable across generations.

## 4. Conclusions and Remarks

Despite the online format imposed by the COVID19 pandemic, the 2020 edition of the Series of Seminars on Advances in Apomixis Research was a great success in strengthening the growing network of scientists involved in apomixis and supported by the European Union H2020 Programme and in providing an opportunity for students and young and senior researchers to share their experience and work.

Biological features of apomicts (e.g., high heterozygosity, polyploidy, resistance to genetic transformation [21]) have long prevented the use of the new “omics” and imaging technologies during their early stages and, most recent advances in the genetics and the molecular control of apomixis have arisen from sexual model species, such as *Arabidopsis*, maize, and rice. Among the best examples are the epigenetic control acting throughout reproductive development [22] and the role of key transcription factors in the acquisition of parthenogenetic competences (i.e., BABY BOOM [23,24]). However, as illustrated by the abstracts reported here, increasing accessibility to modern analytical tools for genome-wide structural and expression studies and to state-of-the-art confocal microscopy is changing profoundly the field by expanding our knowledge about the structure and the functioning of natural apomicts genomes, and by providing novel candidate functions and pathways for apomixis. Results are expected to fuel our understanding of the reproductive biology of apomicts and, therefore, they could contribute efficiently to fine-tune synthetic paths or to improve plant breeding procedures. The main perspective of the MAD project is to resolve candidate mechanisms involved in the regulation of the developmental shift driving sexual reproduction into apomixis through effective sharing of biological resources, expertise, and knowledge gathered from 12 laboratories internationally recognized for their contribution to apomixis research.

## Figures and Tables

**Table 1:** Sexual and apomictic systems studied in Mechanisms of Apomictic Development (MAD).

Species	Status (See Figure 1C)	Available Resources
*Arabidopsis thaliana*, Rice, maize	Sexual model species	Genetic stocks (mutants, reporter lines), transcriptomic resources
*Boechera holboellii*	Apomictic relative of *A. thaliana* (diplospory)	Germplasm collection, genomic, and transcriptomic resources, candidate genes
*Hypericum perforatum*	Apomictic species (apospory)	Transcriptomic resources, candidate genes
*Paspalum* ssp. (Figure 1A)	Apomictic subtropical forage grasses (apospory)	Germplasm collection, genomics, and transcriptomic resources, linkage maps, candidate genes, detailed reproductive calendars, genetic transformation
*Eragrostis curvula* (Figure 1B)	Apomictic subtropical forage grass (diplospory)	Genomics and transcriptomic resources, linkage maps, candidate genes

## Data Availability

No new data were created or analyzed in this study. Data sharing is not applicable to this article.

## Supplementary Materials

The following are available online at https://www.mdpi.com/2223-7747/10/3/565/s1, Table S1: MAD kick-off meeting participants.

## References

[1] Thiault L., Mora C., Cinner J.E., Cheung W.W.L., Graham N.A.J., Januchowski-Hartley F.A., Mouillot D., Sumaila U.R., Claudet J. (2019). Escaping the perfect storm of simultaneous climate change impacts on agriculture and marine fisheries. Sci. Adv..

[2] Steinwand M.A., Ronald P.C. (2020). Crop biotechnology and the future of food. Nat. Food.

[3] Foley J.A., Ramankutty N., Brauman K.A., Cassidy E.S., Gerber J.S., Johnston M., Mueller N.D., O’Connell C., Ray D.K., West P.C. (2011). Solutions for a cultivated planet. Nature.

[4] Jacquier N.M.A., Gilles L.M., Pyott D.E., Martinant J.P., Rogowsky P.M., Widiez T. (2020). Puzzling out plant reproduction by haploid induction for innovations in plant breeding. Nat. Plants.

[5] Vijverberg K., Ozias-Akins P., Schranz M.E. (2019). Identifying and engineering genes for parthenogenesis in plants. Front. Plant Sci..

[6] Nogler G.A. (1984). Gametophytic Apomixes.

[7] León-Martínez G., Vielle-Calzada J.P. (2019). Apomixis in flowering plants: Developmental and evolutionary considerations. Curr. Top. Dev. Biol..

[8] Pennizi E. (2010). Sowing the seeds for the ideal crop. Science.

[9] Lenaerts B., Collard B.C.Y., Demont M. (2019). Review: Improving global food security through accelerated plant breeding. Plant Sci..

[10] Sailer C., Schmid B., Grossniklaus U. (2016). Apomixis allows the transgenerational fixation of phenotypes in hybrid plants. Curr. Biol..

[11] Hanna W.W., Bashaw E.C. (1987). Apomixis: Its Identification and Use in Plant Breeding. Crop Sci..

[12] Vielle Calzada J.P., Crane C.F., Stelly D.M. (1996). Apomixis: The asexual revolution. Science.

[13] Ozias-Akins P., Conner J.A. (2020). Clonal Reproduction through Seeds in Sight for Crops. Trends Genet..

[14] Koltunow A.M., Grossniklaus U. (2003). Apomixis: A developmental perspective. Annu. Rev. Plant Biol..

[15] Grimanelli D., Leblanc O., Perotti E., Grossniklaus U. (2001). Developmental genetics of gametophytic apomixis. Trends Genet..

[16] Ozias-Akins P., van Dijk P.J. (2007). Mendelian genetics of apomixis in plants. Annu. Rev. Genet..

[17] Nakajima K. (2018). Be my baby: Patterning towards plant germ cells. Curr. Opin. Plant Biol..

[18] Pinto S.C., Mendes M.A., Coimbra S., Tucker M.R. (2019). Revisiting the female germline and its expanding toolbox. Trends Plant Sci..

[19] Leblanc O., Grimanelli D., Hernandez-Rodriguez M., Galindo P.A., Soriano-Martinez A.M., Perotti E. (2009). Seed development and inheritance studies in apomictic maize-Tripsacum hybrids reveal barriers for the transfer of apomixis into sexual crops. Int. J. Dev. Biol..

[20] Singh M., Conner J.A., Zeng Y.-J., Hanna W.W., Johnson V.E., Ozias-Akins P. (2010). Characterization of apomictic BC7 and BC8 pearl millet: Meiotic chromosome behavior and construction of an ASGRcarrier. chromosome-specific library. Crop Sci..

[21] Ortiz J.P.A., Pupilli F., Acuña C.A., Leblanc O., Pessino S.C. (2020). How to Become an Apomixis Model: The Multifaceted Case of *Paspalum*. Genes.

[22] Grimanelli D. (2012). Epigenetic regulation of reproductive development and the emergence of apomixis in angiosperms. Curr. Opin. Plant Biol..

[23] Conner J.A., Ozias-Akins P. (2017). Apomixis: Engineering the Ability to Harness Hybrid Vigor in Crop Plants. Methods Mol. Biol..

[24] Khanday I., Skinner D., Yang B., Mercier R., Sundaresan V. (2019). A male-expressed rice embryogenic trigger redirected for asexual propagation through seeds. Nature.

