# Supplementary material for: Proceedings of the 7th Series of Seminars on Advances in Apomixis Research
Source: Plants (Basel). 2021 Mar 17;10(3):565. doi: 10.3390/plants10030565 (PMC8002402; doi:10.3390/plants10030565)
Supplement: Supplementary file 1 [file plants-10-00565-s001.pdf]

Table S1. MAD kick-off meeting participants.

Name, Surname	Affiliation
Albertini, Emidio	University of Perugia, Italy
Autran, Daphné	Institut de Recherche pour le Développement, France
Baroux, Celia	University of Zürich, Department of Plant and Microbial Biology, Switzerland
Belfiori, Beatrice	Institute of Biosciences and BioResources, National Research Council, Italy
Belluci, Michele	Institute of Biosciences and BioResources, National Research Council, Italy
Caccamo, Mario	National Institute for Agricultural Biology, Crop Bioinformatics group, UK
Carballo, José	Centro de Recursos Naturales Renovables de la Zona Semiárida, Argentina
Colombo, Lucia	University of Milan, Italy
Colono, Carolina	Instituto de Investigaciones en Ciencias Agrarias de Rosario, Argentina
Consonni, Gabriela	University of Milan, Italy
Da Marchis, Francesca	Institute of Biosciences and BioResources, National Research Council, Italy
Delgado, Luciana	Instituto de Investigaciones en Ciencias Agrarias de Rosario, Argentina
Echenique, Viviana	Centro de Recursos Naturales Renovables de la Zona Semiárida, Argentina
Espinoza, Francisco	Instituto de Botánica del Nordeste, Argentina
Gallardo, Jimena	Centro de Recursos Naturales Renovables de la Zona Semiárida, Argentina
Gallo, Cristian	Centro de Recursos Naturales Renovables de la Zona Semiárida, Argentina
Garbus, Ingrid	Centro de Recursos Naturales Renovables de la Zona Semiárida, Argentina
Gillmor, Stewart	CINVESTAV, Laboratorio Nacional de Genómica para la Biodiversidad, Mexico
Grossniklaus, Ueli	University of Zürich, Department of Plant and Microbial Biology, Switzerland
Leblanc, Olivier	Institut de Recherche pour le Développement, France
Lejbowicz, Maïa	Institut de Recherche pour le Développement, France
Marconi, Gianpiero	University of Perugia, Italy
Mendes, Marta	University of Milan, Italy
Terzaroli, Nicolo	University of Perugia, Italy
Ortiz, Juan Pablo	Instituto de Investigaciones en Ciencias Agrarias de Rosario, Argentina
Palumbo, Fabio	University of Padova, Italy
Pasten, Cielo	Centro de Recursos Naturales Renovables de la Zona Semiárida, Argentina
Pessino, Silvina	Instituto de Investigaciones en Ciencias Agrarias de Rosario, Argentina
Petrella, Rosanna	University of Milan, Italy
Podio, Maricel	Instituto de Investigaciones en Ciencias Agrarias de Rosario, Argentina
Pupilli, Fulvio	Institute of Biosciences and BioResources, National Research Council, Italy
Rubini, Andrea	Institute of Biosciences and BioResources, National Research Council, Italy
Selva, Juan Pablo	Centro de Recursos Naturales Renovables de la Zona Semiárida, Argentina
Siena, Lorena	Instituto de Investigaciones en Ciencias Agrarias de Rosario, Argentina
Stein, Juliana	Instituto de Investigaciones en Ciencias Agrarias de Rosario, Argentina
Tucker, Matthew	University of Adelaide, School of Agriculture, Food and Wine, Australia
Zappacosta, Diego	Centro de Recursos Naturales Renovables de la Zona Semiárida, Argentina
